# Motor planning of goal-directed action is tuned by the emotional valence of the stimulus: a kinematic study

**DOI:** 10.1038/srep28780

**Published:** 2016-07-01

**Authors:** P. O. Esteves, L. A. S. Oliveira, A. A. Nogueira-Campos, G. Saunier, T. Pozzo, J. M. Oliveira, E. C. Rodrigues, E. Volchan, C. D. Vargas

**Affiliations:** 1 Laboratório de Neurobiologia II, Instituto de Biofísica Carlos Chagas Filho, Universidade Federal do Rio de Janeiro, Rio de Janeiro, Brasil; 2 Programa de Pós-graduação em Ciências da Reabilitação – Centro Universitário Augusto Motta, Rio de Janeiro, Brasil; 3 Departamento de Fisiologia, Instituto de Ciências Biológicas, Universidade Federal de Juiz de Fora, Juiz de Fora, Brasil; 4 Laboratório de Cognição Motora, Instituto de Ciências Biológicas, Universidade Federal do Pará, Belém, Brasil; 5 INSERM - U1093 Cognition, Action, et Plasticité Sensorimotrice, Campus Universitaire, UFR STAPS, Dijon, France

## Abstract

The basic underpinnings of homeostatic behavior include interacting with positive items and avoiding negative ones. As the planning aspects of goal-directed actions can be inferred from their movement features, we investigated the kinematics of interacting with emotion-laden stimuli. Participants were instructed to grasp emotion-laden stimuli and bring them toward their bodies while the kinematics of their wrist movement was measured. The results showed that the time to peak velocity increased for bringing pleasant stimuli towards the body compared to unpleasant and neutral ones, suggesting higher easiness in undertaking the task with pleasant stimuli. Furthermore, bringing unpleasant stimuli towards the body increased movement time in comparison with both pleasant and neutral ones while the time to peak velocity for unpleasant stimuli was the same as for that of neutral stimuli. There was no change in the trajectory length among emotional categories. We conclude that during the “reach-to-grasp” and “bring-to-the-body” movements, the valence of the stimuli affects the temporal but not the spatial kinematic features of motion. To the best of our knowledge, we show for the first time that the kinematic features of a goal-directed action are tuned by the emotional valence of the stimuli.

The basic underpinnings of homeostatic behavior include motor interactions with emotion-laden objects. Compelling evidence shows that in humans emotion-laden contexts affect motor output^1^,^2^,^3^,^4^,^5^,^6^. Employing readiness potential, an electrophysiological marker of motor preparation, our group demonstrated that bringing unpleasant stimuli towards the body results in a higher cost compared to pleasant stimuli, indicating that motor planning encompasses an estimate of the action value (costs and gains)^4^. In such a context, preparing to interact with pleasant stimuli would recruit pre-set approach-like motor repertoires^4^. Conversely, when preparing to interact with unpleasant stimuli, the discrepancy between the required action and the object’s aversiveness would result in a broader mobilization of neural resources^4^,^5^.

The kinematic parameters of a given action have long been postulated to reflect the content of the motor plan^7^,^8^,^9^,^10^,^11^,^12^,^13^. Kinematic invariants of actions can be captured from different individuals performing the same task such as, for instance, picking up a glass from a table. Indeed, the kinematic features of the upper-limb during goal-directed actions have been proven to reflect the participant’s intentions (grasping or pointing at something; grabbing to throw, lifting or fitting something)^13^. The same holds for the intrinsic characteristics of the object (geometry, texture, weight, size, and shape) with which the agent interacts^14^. Besides, social intentions and their motoric components translate into specific kinematic features^15^,^16^,^17^,^18^.

Since the kinematic features of goal-directed actions reflect motor planning^10^ and are modulated by intentions^13^,^14^,^15^,^16^,^17^,^18^, the kinematics of actions directed towards unpleasant stimuli could reflect action costs, whereas those directed towards pleasant stimuli could reveal facilitation. Such a result would be in agreement with the idea that motor plans encompass the costs and gains (value)^4^,^19^ of a given action. To address this issue we employed a paradigm consisting of an analysis of real movements of grasping emotion-laden stimuli and bringing them toward the body. During reach-to-grasp (phase A), when the participant interacted with the stimulus by the first time, our conjecture was that the stimuli affective load should be made evident in the measured kinematic parameters. Crucially, we hypothesized that action facilitation would be especially manifested during the bring-to-the-body period (phase B), in congruence with the final goal of the action.

## Results

### Affective Rating

**Figure 1:**
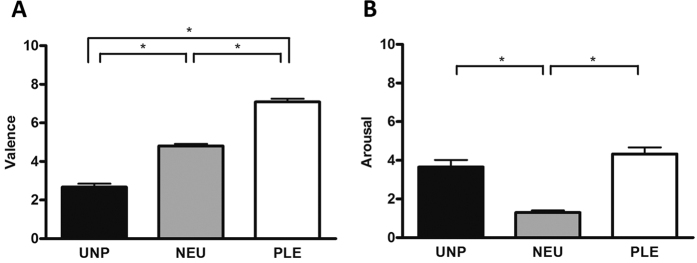
Affective Rating. Valence (**A**) and Arousal (**B**). Means and Standard Error, n = 20. Unpleasant, Neutral and Pleasant Stimuli. *p < 0.01.

The affective rating of emotion-laden stimuli collected from twenty participants tested by means of repeated measures Anova with the emotional category as the factor revealed a main effect of valence [F (2, 38) = 226,09, p < 0.0001)] (Fig. 1A). The same statistical approach also yielded a main effect of arousal [F (2, 38) = 25,87, p < 0.0001)] (Fig. 1B). Post hoc analysis of the valence dimension showed higher scores for pleasant (7.09 ± 0.16, Mean and SE) compared to neutral (4.8 ± 0.11) and unpleasant (2.67 ± 0.17) stimuli. Furthermore, participants rated the unpleasant stimuli with lower scores compared to the neutral ones. In the arousal dimension participants gave similar scores for unpleasant (3.65 ± 0.37) and pleasant (4.33 ± 0.34) stimuli and both were higher than the neutral (1.30 ± 0.10) ones.

### Kinematics Parameters

The effect of emotion over a goal-directed action was verified by means of a three-way repeated measures Anova with the phases (reach-to-grasp and bring-to-the-body), valence (unpleasant, neutral and pleasant) and blocks (1, 2 and 3) as independent factors for each kinematics parameter.

### Movement Time

**Figure 2:**
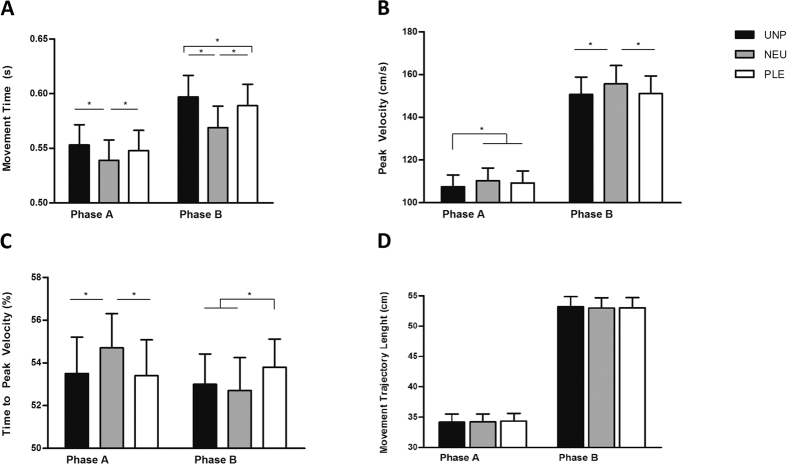
Kinematics of reach-to-grasp (phase A) and “Bring-to-the-body” (phase B). (**A)** Movement Time; (**B)** Peak Velocity; (**C)** Time to Peak Velocity; (**D)** Movement Trajectory Length. *p < 0.05.

A significant interaction between phase (reach-to-grasp and bring-to-the-body) and valence (unpleasant, neutral and pleasant) [F (2, 40) = 6,27, p = 0.004] was found in relation to the length of time of the movement. The Movement Time was longest for reach-to-grasp unpleasant and pleasant stimuli in comparison to neutral stimuli. Additionally, the Movement Time was longer to bring-to-the-body unpleasant stimuli compared to the pleasant and neutral stimuli, and longer for bringing pleasant stimuli toward the body than neutral stimuli (Fig. 2A). A main effect for phase [F (1, 20) = 5,33, p = 0.032] and for valence [F (2, 40) = 30,17, p < 0.001] was also revealed. Post-hoc comparisons showed that the reach-to-grasp phase was shorter than the bring-to-the-body phase and unpleasant stimuli lasted more than pleasant and neutral ones. Furthermore, the duration was longer for pleasant than neutral stimuli. No other effects were observed for this parameter (See Table 1).

### Peak Velocity

Repeated measures Anova performed on Peak Velocity showed significant interaction between phase and valence [F(2, 40) = 4,0822, p = 0.024]. Participants had lower Peak Velocity values to reach-to-grasp (Phase A) unpleasant stimuli than pleasant and neutral stimuli. Moreover, during the bring-to-the-body phase (Phase B), Peak Velocity was lower for pleasant and unpleasant stimuli than neutral stimuli. A main effect of phase F(1, 20) = 49,622, p < 0.001] and of valence F(2, 40) = 10,744, p < 0.001] was also revealed. Post-hoc comparison showed that Peak Velocity was lower during Phase A when compared to Phase B. Additionally, Peak Velocity was lower for pleasant and unpleasant than neutral stimuli. No other effects were observed for this parameter (See Table 1 and Fig. 2B).

### Time to Peak Velocity

**Figure 3:**
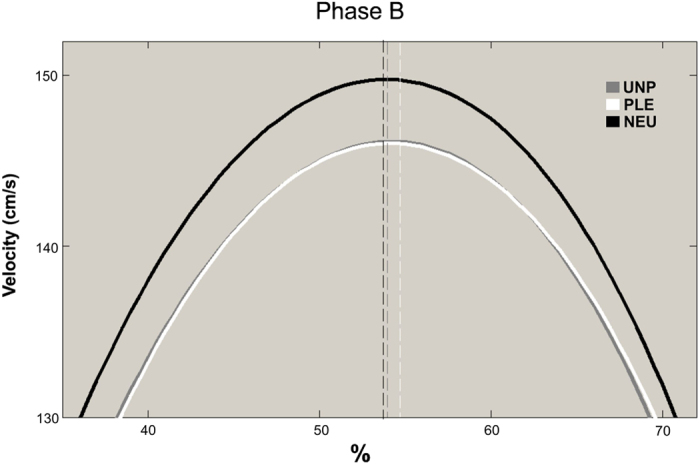
Tangential Velocity Profile. Mean of three blocks for 21 participants in phase B (bring-to-the-body) for each emotional category. Unpleasant is in black, neutral in grey, and pleasant in white.

A significant interaction between phase and valence was found for the Time to Peak Velocity parameter [F(2, 40) = 6,29, p = 0.004]. Post-hoc comparisons showed that the deceleration phase was longer when reach-to-grasp pleasant and unpleasant stimuli when compared to neutral ones. Interestingly, during the bring-to-the-body phase, deceleration was shorter for pleasant stimuli when compared to unpleasant and neutral stimuli (Figs 2C and 3). No other effects were observed for this parameter (See Table 1).

### Movement Trajectory Length

No significant interaction between phase and valence was found for the Movement Trajectory Length parameter [F (2, 40) = 1,28, p = 0.288] (Fig. 2D and Table 1). A main effect was found for Phase [F (1, 20) = 77,23, p = 0.0001]. The reach-to-grasp length was shorter than the bring-to-the-body one. No other effects were observed for this parameter (See Table 1).

## Discussion

In the present study we aimed to evaluate whether the kinematic features of a goal-directed action are affected by the emotional valence of the stimuli. We employed a real interaction paradigm in which volunteers were asked to physically interact with stimuli classified as unpleasant, neutral and pleasant. The kinematic analysis revealed that the Time to Peak Velocity, Movement Time and Peak Velocity parameters were modulated by the valence of the stimuli, whereas there was no emotional category effect in the trajectory length of the movement. The valence effects reflected in Time to Peak Velocity, Movement Time and Peak Velocity can thus not be attributed to differences in trajectory length.

Previous work showed that kinematics is affected by the emotional context induced by valence-laden pictures^20^,^21^. In the present study, the source of emotion is inherent to the goal of action. To the best of our knowledge, we show for the first time that the temporal features of motion kinematics are tuned by the emotional valence of the stimulus with which one is about to interact, thus strengthening the premise that emotion affects motor planning.

The participants spent more time in reach-to-grasp and bring-to-the-body pleasant and unpleasant stimuli compared to neutral stimuli. Moreover, participants attained lower Peak Velocity values to reach-to-grasp unpleasant stimuli than pleasant and neutral ones. Interestingly, during the bring close to the body phase, Movement Time was even longer for unpleasant stimuli when compared with pleasant and neutral ones. The Peak Velocity parameter followed a similar trend during this phase, being lower for pleasant and unpleasant stimuli compared to neutral ones. In the same vein, it has been shown that viewing unpleasant pictures promotes the slowing of reaction times^1^,^3^, modulates force production^2^ and reduces body sway^22^,^23^. Thus, our interpretation is that Movement Time and Peak Velocity parameters might reflect a global affective load effect, being specially sensitive to the stimuli unpleasantness.

Analysis of the Time to Peak Velocity showed a longer deceleration time (shorter Time to Peak Velocity) in the reach-to-grasp phase (phase A) in the valence-laden conditions compared to the neutral condition. At first glance, this result could be interpreted as a facilitation effect for the neutral condition. Indeed, a longer deceleration time could indicate a higher demand to perform the task^13^,^28^. When the participants have to first interact with an emotion-laden stimulus, a high recruitment of affective resources is expected to occur^24^,^25^. Furthermore, emotional stimuli catch more attention^1^ and promote a more careful evaluation to define their aversiveness or pleasantness^26^, hence the apparent facilitation effect (shorter deceleration time) for the neutral stimuli. Crucially, for this parameter the deceleration time was shorter when pleasant as compared to unpleasant and neutral stimuli were brought close to the body. During this phase, when the participant achieves the goal of the motor plan, he has already identified the stimuli valence. If a longer deceleration time indicates higher demand to perform the task^13^,^27^, shorter deceleration time could correspond to higher easiness in achieving the purpose of the task. Thus, the pleasantness of the stimulus would in fact *facilitate* action implementation.

In accordance with this hypothesis, the velocity of saccade movements has been shown to be greater when performed towards human faces^19^, thus valence-laden, in contrast to an image of a neutral object. Also, higher Peak Velocity and lower Time to Peak Velocity values were described when participants were asked to reach-to-grasp towards another person compared to a single-agent condition^27^. Likewise, lower readiness potential amplitudes have been found to precede the grasping of pleasant stimuli compared to unpleasant and neutral ones^4^. The authors proposed that the pleasantness of the stimuli recruited preset approach-like circuits in the brain, making the action less costly. In another line of evidence, lower readiness potentials preceded grooming actions performed in a pleasant social bonding context^6^. Finally, lower corticospinal excitability was found during reach-to-grasp pleasant stimuli compared to unpleasant and neutral ones^5^. The authors argued that the pleasant stimuli triggered an urge to move that required greater suppression, reflecting enhanced control preceding actions towards those stimuli. Applied to the present results, we propose that the implied intention^13^,^14^,^15^,^16^,^17^,^18^ embedded in bring-to-the-body a pleasant stimulus matched with preset approach-like motor repertoires yield a shorter deceleration time in the pleasant condition compared to the unpleasant and the neutral conditions.

The lack of difference in time to peak velocity found between unpleasant and neutral stimuli when they were brought close to the body may be explained as follows: preset withdrawal programs triggered by object aversiveness would compete with the implementation of an imposed bring close to the body action, resulting in the mobilization of more neural resources and thus in higher cost^4^,^5^. This incongruence did not translate however into any modulation over action kinematics, suggesting that bringing an unpleasant stimulus towards the body was implemented as if it was a neutral stimulus. In other words, our conjecture is that, to comply with the experimenter’s instructions and interact with the unpleasant stimulus, the participants might implicitly reduce its unpleasantness as if it was a neutral stimulus (se also^28^,^29^ for an attenuation effect of unpleasant stimuli induced by their reappraisal).

In conclusion, the kinematics of reach-to-grasp and bring-to-the-body movements is affected by the emotional valence of the stimulus to which one interact. Shorter deceleration time indicates higher easiness in bringing pleasant stimuli close to the body whereas the lack of any differences with the neutral and unpleasant stimuli might result from having to comply with the demand of performing an otherwise unwanted action. Kinematics thus seems to embody the motor intentions that relate to the value (costs and gains) of the stimuli with which one is interacting.

## Methods

### Participants

Twenty-five right-handed male students aged 21–36 years old (27.71 ± 4.12) participated in this study. A single gender sample was chosen because emotionally laden stimuli categorization is gender specific^30^,^31^. Participants reported having no neurological or neuropsychiatric disease. Written informed consent was provided by each participant. All experimental protocols were approved by the local Ethics Committee (CEP n°092376/2013 - 5257 Hospital Universitário Clementino Fraga Filho/UFRJ). The methods were carried out in accordance with the approved guidelines of the Hospital. Handedness was assessed with the Edinburgh handedness inventory^32^.

### Stimuli selection

A set of 60 emotional-laden objects was placed inside identical transparent cylinders to facilitate uniform grip and was balanced in weight. A total of 39 emotional-laden stimuli (thirteen unpleasant, neutral and pleasant) were selected using a Self-Assessment Scale (SAM)^33^ from a previous study^4^. The objects inside the cylinders were also evaluated based on their dimensions in a behavioral test in which participants judged the type of the grasp they would employ to interact with each stimuli^4^. The type of the grasp (precision grasp or whole grasp) was balanced among the three emotional categories.

Accommodated inside each transparent cylinder, the *unpleasant* stimuli were a chicken gizzard, a cake with hair, artificial vomit, a preserved cockroach, artificial excrement, preserved rotten food, a bluebottle on a biscuit, a preserved dead rat, a rotten artichoke, a preserved chicken foot, an artificial spider, an artificial snake and a preserved fish eye; the *neutral* ones were adhesive tape, a pencil sharpener, a crumpled paper ball, silver paper clips, binder clips, a sponge, a glue stick, a piece of plastic bag, an alkaline battery, cotton balls, pieces of colored wire, spun wool and a strip of staples; and the *pleasant* stimuli were a chocolate candy, chocolate tablet, money, a wrapped condom, mobile phone, some soccer cards, two toys cars, marbles, a gold trophy, a ball, a television remote control, an MP3 player and wrist watch.

### Procedure

**Figure 4:**
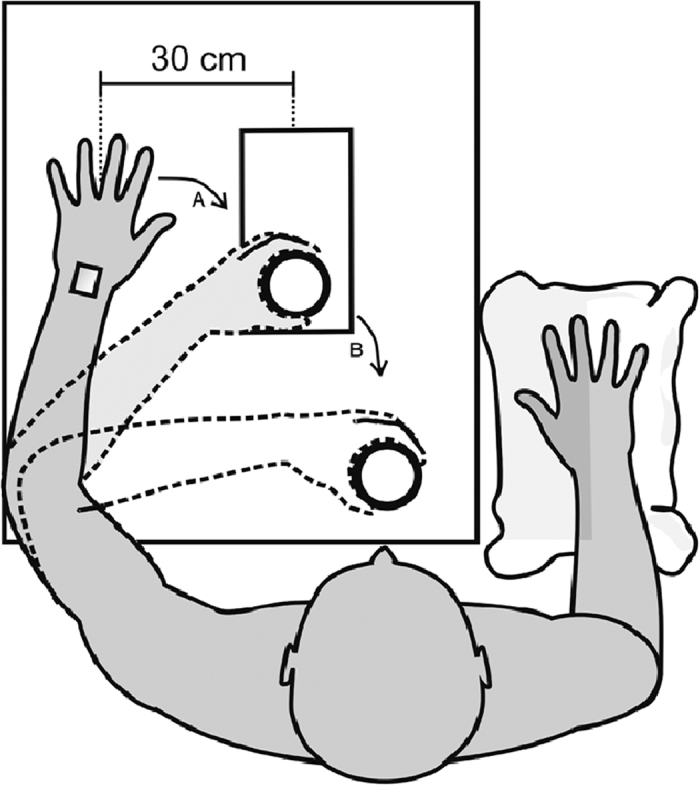
Experimental setup. The reach-to-grasp (**A**) and “bring-to-the-body” (**B**) phases of the action are indicated, as well as the initial distance between the hand and the stimulus. The white rectangle indicates the position of the mobile sensor on the participant´s wrist.

The experiment was conducted in a sound-attenuated room under ambient light. The participant was asked to sit in a comfortable chair facing a table on which the stimuli (identical transparent cylinders containing the emotion laden object) were presented, one at a time, by an experimenter seated behind a black curtain in front of each participant. The stimulus was presented on a mobile wooden tray with a holder for the stimulus, fixed 30 cm from the hand of the participant (Fig. 4). The experimenter withdrew the tray to change the stimulus after each trial. Each stimulus was presented once in a randomized block of 39 trials. The experiment comprised a total of four blocks. The first one was a training block to familiarize participants with the experimental task. Approximately 3 minutes of rest between blocks were given to the participant. The total duration of the experiment was about fifty minutes.

Kinematic data collection was performed *in tandem* with a readiness potential experiment^4^. Participants performed the task with their left hand, as stronger readiness potential negativity was reported for movements with the non-dominant left hand^4^. At the beginning of each trial, the participants were instructed to rest their left hand on the table on a load cell and focus on the point where the stimulus would appear. Upon the stimulus presentation they were instructed to wait for a few seconds and, whenever they felt ready, grasp the cylinder (containing the emotional-laden object) with their left hand and bring it close to their chest. After that, they returned the cylinder to the tray and repositioned their hand on the load cell in the initial position (Fig. 4). A training session ensured that participants waited approximately 3 s to initiate the task. This was accomplished by giving verbal feedback during the training without explicit information about the desired interval.

Each participant was also instructed to keep his left elbow in contact with the table and to avoid making any other movement during the experiment. The right arm, not involved in the task, rested on a pillow during the experimental session. To motivate participants to pay attention to the stimuli, they were asked to carefully observe each stimulus in order to be able to identify, after the experiment, which stimuli they had seen during the experimental session. At the end of the experiment all participants except one evaluated each stimulus in both dimensions of emotion (valence and arousal)^4^ using the Self-Assessment Scale (SAM)^33^.

### Kinematic recording

An electromagnetic tracking device, the Polhemus Fastrak (SPACE FASTRAK, Colchester, VT, USA) was used to record the left wrist position in three-dimensional coordinates. A stationary transmitter was fixed 30 cm in front of the participant and established the global coordinate system. A mobile sensor was fixed with an adhesive tape on the dorsal aspect of the left wrist at the intersection point between the styloid process of the ulna and the middle finger. Data acquisition was synchronized with the stimulus presentation. Real-time 3-D position of the left wrist was tracked at 100 Hz during the execution of the task, permitting off-line calculation of wrist displacement over time and the velocity profile.

### Data analysis

Data analysis was made offline using Matlab software (Mathworks, USA). A 3-D reconstruction of the wrist trajectory was performed and its tangential velocity profile was calculated and filtered with a fifth-order low pass filter at 10Hz. The action was divided in two phases: (A) reach-to-grasp and (B) bring-to-the-body. A Matlab script was used to determine the onset of the reach-to-grasp phase, calculated as five percent of the first peak of velocity^14^. The beginning of phase B was determined as the lowest velocity profile value between phases A and B. The end of the bring-to-the-body phase (phase B) was defined as the smallest value after its peak. The tangential velocity profile of each separated phase was time-normalized by a linear interpolation of 200 points. A second Matlab script was designed to detect discrepant shapes of the velocity profile. The two phases of all trials were plotted together so that discrepant shapes from the participant´s median value for each condition and each phase could be marked and then excluded through visual inspection blind to the valence condition. If one outlier phase was beyond the average the whole trial was discarded from the analysis. Less than 10% of trials were excluded using these criteria. Four participants were excluded from the kinematic analysis due to technical problems in data collection.

The following parameters were calculated for the remaining 21 participants: movement time (MT), peak velocity (PV), time to peak velocity (TPV) and movement trajectory length (MTL) for reach-to-grasp and bring-to-the-body phases. Movement time was defined as the time interval between the onset and offset of each phase. Movement trajectory length was determined as the distance traveled for each participant in each phase. Peak Velocity corresponds to the maximal velocity attained for each participant in each phase. Time to peak velocity represents the timing of motion, i.e.: the proportion between the duration of the acceleration and the deceleration times^12^, that is, the ratio between acceleration time (AT) and movement time (MT). This index indicates how long the acceleration time of a movement lasted with respect to the total duration of the movement. A ratio greater than 0.5 indicates that the deceleration time is shorter than acceleration time. This parameter indicates the motor system strategy to perform the action, since the deceleration time increases with task demand^13^.

### Statistical Analyses

A Three-way repeated measures Anova was performed using Statistica 7 software, with phases (reach-to-grasp and bring-to-the-body), valence (unpleasant, neutral and pleasant) and blocks (1, 2 and 3) as independent variables for each kinematics parameter. Post hoc analysis was assessed using Duncan’s test whenever a significant effect was found, i.e. both on main effects and on interactions. One-way repeated measures Anova was run for the valence and arousal ratings separately having the emotional category (unpleasant, neutral and pleasant) as within factor. Duncan’s post-hoc analysis was employed on any significant effect that emerged from the ANOVA. The level of significance was set to 0.05.

We introduced “block” as a factor in the ANOVA to guarantee that there would not be any learning or habituation effect with respect to the emotional valence of the stimuli^34^.

## Additional Information

**How to cite this article**: Esteves, P. O. *et al*. Motor planning of goal-directed action is tuned by the emotional valence of the stimulus: a kinematic study. *Sci. Rep.* **6**, 28780; doi: 10.1038/srep28780 (2016).

## Figures and Tables

**Table 1:** Mean and Standard Error for each parameter for reach-to-grasp (phase A) and bring-to-the-body (phase B).

Movement Time (s)				
Valence	Mean	Standard Error	Mean	Standard Error
	PHASE A		PHASE B	
Unpleasant	0.553	0.019	0.597	0.020
Neutral	0.540	0.019	0.569	0.020
Pleasant	0.548	0.018	0.589	0.020
Statistics Values	[F(2,40) = 6,27, p = 0.004]			
**Peak Velocity (cm/s)**				
Unpleasant	107.530	5.439	150.715	8.104
Neutral	110.273	5.944	155.657	8.606
Pleasant	109.203	5.621	151.104	8.248
Statistics Values	[F(2, 40) = 4,082, p = 0.024]			
**Time to Peak Velocity (%)**				
Unpleasant	53.465	1.669	53.015	1.412
Neutral	54.733	1.676	52.694	1.314
Pleasant	53.361	1.587	53.838	1.540
Statistics Values	[F(2,40) = 6,29, p = 0.004]			
**Movement Trajectory Length (cm)**				
Unpleasant	34.163	1.337	53.203	1.655
Neutral	34.195	1.328	52.995	1.677
Pleasant	34.356	1.253	53.035	1.670
Statistics Values	[F(2,40) = 1,283, p = 0.288]			
